# Supplementary material for: Chromothripsis during telomere crisis is independent of NHEJ, and consistent with a replicative origin
Source: Genome Res. 2019 May;29(5):737–49. doi: 10.1101/gr.240705.118 (PMC6499312; doi:10.1101/gr.240705.118)
Supplement: Supplemental Material [file supp_gr.240705.118_Supplemental_file_1.zip › contigs/annotated_contigs/DB73/contig.2.DB73_length_588_mean_cov_6.80612244898.docx]

**DB73_length_588_mean_cov_6.80612244898**

```
ATATGCAAGGACATTTGCCACTTCTACTCTTCTAAACCCAATTTATCAATCTGCCAGTCCATTCCCAATGTCATCCCAGAAACTCATAG
 >chr16:59291572-59291904 + E=4e-185
AACACTCCTCAGAACACTTGTCAAATGCTTTTCCTGCATCTATCGAGATGATCATTTGATTTATGTCTGTTATTTTATTAATATGGTAT

ATTACATATTGAGATTTGAACGTCAAATCAACCTTATATTTCTGGATAAGCCCAACATGGCATAGTGTATAATCATTTTTATATGTCCC

TGAATTCAGTTTGTGATTGTTAAGTATTTTTGTGGTTACGTTCATAAGAAATATGGATTTTCTT|T|CTGATTCTTCTAAAGAGTTATG
                                                                 >chr16:59286728-592869
TATTTCTGAGATGAGTTTTTTTTATTTGTTGATTGCCAACAAGCTGCAAACAATAATATGTAAAGACACCTGAGTTAATTTGAGTGGCT
85 - E=6e-143
TTTCCAGGGCTTCTCAAGCTTTAATGTGCATATGGTTCACCTGAGGATTGTGTTACACTTCAGATTCTGATTCAGTAGGTCTGGAATGG

GACCTAAGAGTATGCATTTCTGATAAGTTGGTCTGCTGTTGATCAGCTCACAAGTT
```
